# Women in family farming: Evidence from a qualitative study in two Portuguese inner regions

**DOI:** 10.3389/fsoc.2022.939590

**Published:** 2022-12-07

**Authors:** Diana Gomes, Miguel Jesus, Rosário Rosa, Cristina Bandeira, Cristina Amaro da Costa

**Affiliations:** ^1^School of Agriculture, Polytechnic Instituto of Viseu, Viseu, Portugal; ^2^Faculty of Arts and Humanities of University of Porto, Porto, Portugal; ^3^National Observatory of Violence and Gender, CICS.NOVA/NOVA Faculty of Social and Human Sciences, Lisbon, Portugal; ^4^Open University of Lisbon, Lisbon, Portugal; ^5^CEF—Centre for Functional Ecology, Science for People and the Planet, University of Coimbra, Coimbra, Portugal; ^6^Women's Alternative and Response Union, Lisbon, Portugal; ^7^CERNAS—IPV Research Centre, Viseu, Portugal

**Keywords:** women farmers, gender equality, feminization, gender division of labor, family farming

## Abstract

The importance of family farming in food systems worldwide is recognized by different international bodies, as well as the leading role played by women and the inequalities they face in this sector of activity. The most recent data from Portugal highlight the importance of this type of agriculture in this Southern European country. In 2019, 68% of the total agricultural workforce in the country was concentrated in family farming, with almost half of them being women. This high permanence of women in agriculture is the result of a long process of feminization on this sector that is similar to other contexts. Despite this strong feminization of family farming, there are few studies that portrait agricultural activity from the women's viewpoint, since the voice of men is always predominant in all references. Based on the exploratory qualitative data from two *focus groups*, carried out in two Portuguese inner regions, we intend to address the perceptions and meanings of a small group of women farmers regarding their activity, the role taken by them in agriculture and the difficulties they experience. Issues such as changes in agriculture and the sexual division of labor will also be addressed in this article. Within these groups, women work in agriculture is perceived as long, solitary and uncertain. Also, the public/private dichotomy is evident, with decision-making and public places dominated by men. A prevalence of the discourse of “masculinization” still exists with certain tasks being attributed to men (e.g., operations with machinery). Younger women (34 and 40 years old) tend to overcome these gender differences choosing agriculture as a profession and healthy and sustainable life for their families.

## Introduction

The economic, environmental, social and cultural importance of family farming was recognized by the Food and Agriculture Organization (FAO) of the United Nations and reflected in the implementation, in 2014, of the International Year of Family Farming. This recognition aimed to put “…family farming at the center of agricultural, environmental and social policies in the national agendas…” (FAO, 2014, 2015) and, later on, by proclaiming the United Nations Decade of Family Farming (2019–2028), as an opportunity to address family farming from a holistic perspective and to contribute to achieve the 2030 Agenda for Sustainable Development (FAO, IFAD, 2019). This recognition reinforces the role of family agriculture in fighting hunger and poverty, in preserving natural resources and the landscape, in improving the quality of life of local and rural communities, and in food security (FAO, 2015; FAO, IFAD, 2019).

Family farming is a concept used to identify agricultural production units that are managed at the family level and relies predominantly on family labor, with a strong relationship between land, labor, and family (Mesquita, 2013; FAO, IFAD, 2019; Costa et al., 2020). The family is simultaneously a production and consumption unit: work is organized by and for the family. Family farming is the most predominant type of agriculture worldwide: it was estimated in 2014 to occupy about 70–80% of the world's agricultural land, account for about 90% of agricultural production in 93 countries, and be the source of 80% of the food consumed that guarantees food for 40% of the world's households (Lowder et al., 2014; FAO, 2015). In Europe, in 2016, about 96% of agricultural land is dedicated to family farming, where about 90% of the farming population works (EU, 2020).

In this context, the role that women play in this sector of agriculture also has been the focus of attention of various international bodies. Women represent, on average, almost half of the agricultural labor force, playing a central role in family farming, not only through their work, but also because of their ancestral knowledge, sustainable management of natural resources, production and conservation of agricultural products, as well as caretakers of children and/or the elderly (FAO, IFAD, 2019). In 2011, in “The State of Food and Agriculture 2010–2011” report, with the subtitle “Women in Agriculture. Closing the gender gap for development,” the role of women in the development of rural areas and the economy, particularly in developing countries (where women occupy 43% of the agricultural workforce), was recognized (FAO, 2011). In the same report, FAO recognized the inequalities and difficulties that women farmers face compared to men farmers in various areas such as access to land, education, markets and services, among others.

In 2016, the European Institute for Gender Equality (EIGE) listed this type of gender inequalities in the agriculture sector, at European level, namely related with women's participation and representation in rural development and in decision-making positions in agriculture (EIGE, 2016). In fact, in the same year, in the European Union only 3 out of 10 agricultural leaders were women (28.7% women in these positions) (EU, 2020).

The invisibility of women farmers and their work in agriculture (EIGE, 2016), as well as the preponderance of male at the center of the discourse in family farming (Whatmore, 1991; Shortall, 1999; Brandth, 2002; Carmo, 2007a; Contzen and Forney, 2017; Shortall et al., 2020), originates a knowledge gap about women roles and importance in family farming. This is specially relevant in countries, such as Portugal, were family farming still represents 94% of the farms, with more than 30% being managed by women (INE, 2021). Furthermore, at the national level, there is no updated and in-depth information about Portuguese women farmers and their contributions at the social, economic and environmental levels. This article aims to give voice to women farmers and provide insight to help other researchers to produce knowledge about women's agricultural work in Portugal, which will contribute to the development of better public policies that respond to the real problems of family farmers and especially women farmers. Thus, we intend to answer the following research questions: (1) How do women farmers perceive their activity in three rural areas of Portugal? (2) What are the role and difficulties that women farmers experience in agriculture? (3) Is there a gender division of labor in family farming?

### Women and family farming in Portugal

Although major transformations have occurred, both in agricultural activity and in rural territories and their communities, over the last decades, the most recent data from Portugal, suggest the importance that family farming maintains in the country (Portela, 1999; Peixoto, 2004; Almeida, 2020).

In 2019, in Portugal about 68% of the agricultural work volume is carried out by the family farming population occupying the 14th place in the European Union (INE, 2021). Only 13.1% of farmers live exclusively from agriculture and ~2/3 occupy < 50% of their working time on the farm. Most farms are managed by individual producers (94.5%), although the number of companies has increased in the last decade (+115.5%). The representativeness of women leading farms is 33.3% (above the EU28 average of 30.1%), but 47.9% of the farm labor force is provided by women (INE, 2021).

The strong feminisation denoted by quantitative data in family farming is not a recent phenomenon in Portugal. The presence of women in agriculture is observed in Portuguese statistics since the second half of the 20th century (Wall, 1986). The statistical information available in the censuses, despite some weaknesses and limitations, suggested a growing female labor force in comparison to the male labor force, in agricultural activity (Rodrigo, 1986). This increase is noted both as individual farmers and unpaid family workers.

This process of feminisation of the agricultural sector dialogued with broader social processes, namely industrialization, and the consequent rural exoduses (Rodrigo, 1986; Wall, 1986). In the case of industrialization, this process caused the displacement of male labor to other areas of activity and made agricultural activity less prestigious and rewarding (Cernea, 1978; Rodrigo, 1986). The rural exoduses are intrinsically linked to the greater opportunity for work abroad and in other professions offered to men (Wall, 1986). In short, these macro-social transformations, allied to micro dynamics within the family, placed the woman as the center of work within family agriculture, in countries such as Portugal (Cernea, 1978; Rodrigo, 1986; Wall, 1986).^1^

Thus, despite the reduction in agricultural assets in both genders “(…) *the regular presence of women in agricultural work is almost always contrasted with an irregular and more or less important male presence*…” (Peixoto, 2004, p. 662). In fact, over the last 30 years old, it has been women who have remained in agriculture the most. National data reveals this trend: while in 1989 there were 501,978 men (84.6% of the total of single farm holders) and 91,870 women leading agricultural holdings (15.5%), 30 years old later 183,916 men (67% of the total of single farm holders) and 90,332 women (33%) remained as managers of their farms (INE, 2021). This variation stands for a less 63.4% for men and less 1.7% for women.

Despite the fact that there are more women remaining in the agricultural activity and that women assuming leadership of their farms have increased, formal leadership positions continue to be mainly “male territory.” In fact, in Portugal, in 2019, around 69% of farm managers were men; women hold only 31% of leading positions, while in 1989, 83.7% of farm managers were men and only 16.3% were women (INE, 2021).

However, this feminization of the agricultural labor force and leading roles has not been sufficient to eliminate gender inequalities in this sector (Lastarria-Cornhiel, 2008). Although this feminization is evident, nationally and internationally, there are few studies that portray agricultural activity from the point of view of women (Whatmore, 1991; Shortall, 1999; Brandth, 2002; Carmo, 2007a; Contzen and Forney, 2017), remaining almost always around male-dominated places of discourse. It is thus important to better understand the issue of gender equality in this specific social context, and to identify factors of inequalities between men and women farmers.

### Gender roles in family farming

With regard to European research on gender roles in agriculture, Sarah Whatmore (2016) outlined some conceptual changes, namely to stop looking at men and women as fixed categories, but rather taking into account the meanings and practices that define and characterize them. The use of gender role theory gave way to gender identity theory, more specifically the representations in daily life of what it means to be a man or a woman farmer, thus giving greater prominence to the roles and tasks performed by them (Brandth, 2002; Whatmore, 2016).

Discourse is one of the most used units of analysis in the literature to understand existing gender inequalities. The results of studies carried out within this scope point to an ever-present patriarchal logic (Shortall, 1999; Haugen et al., 2015). Brandth (2002), carried out a study on gender identity in European family farming, and identified that the positions of male and female farmers portrayed in the literature were linked to 3 types of discourses: “*the discourse of the family farm*,” “*masculinisation,”* and “*detraditionalization and diversity*.” In the first discourse, the male farmer is portrayed as the “public figure” of the family, and the woman is almost in a secondary position, not participating in events outside the family circle. Land ownership is seen as something that should pass from father to son, while women see their access limited and it is only possible to obtain the title of land owner through widowhood or marriage, i.e., the title of land owner never comes from the woman's choice, but from marriage. This type of discourse highlights the patriarchal character as one of the primary characteristics of family farming.

The second discourse focuses on the process of masculinisation of agriculture, i.e., the transformation of agriculture into a markedly male area of work. Family farming has a strong masculine connotation, as it is the man who controls and manages the family business, so when one thinks of a farmer there is a tendency to imagine a physically strong male individual (Shortall, 1999; Brandth, 2002). The process of industrialization of agriculture was a period where women lost part of the central role they had. A good example of this is the mechanization of milk production: “*Milking used to be an important task for women, but in the mid 1900s when the milking machine was introduced, it became just as much an area of work for men, and thus women were forced into a secondary position, or into a position of housewife*” (Lastarria-Cornhiel, 2008, p. 188). Agriculture followed the global process of industrialization and, with it, an increase demand for more skills from farmers overcome, with men being the first one to acquire them, leaving women in a secondary role (ibid.).

The third and last type of discourse portrays a shift toward the empowerment of women farmers. Research on women farmers in Europe reveals the occupation of new positions such as “(…) *farmer, housekeeper, business secretary, farm assistant, agricultural worker, off-farm income earner* (…)” (EIGE, 2016, p. 192). The positions that women farmers occupy nowadays are changing: according to Haugen and Brandth (1994), younger women farmers choose agriculture as a profession and, calling themselves professional women farmers, seek to acquire skills that give them autonomy in farm management, such as driving agricultural machinery (a task usually dominated by men).

Gomes et al. made a more recent analysis of the scientific production on women in rural contexts and concludes that the current thematic focus can be grouped into two subthemes, namely the “… *gender relations and rural spaces that encompass discussions focused on: (a) class relations; (b) gender relations; (c) care and health; (d) right to access to water; (e) body and sexuality (…) and gender and new ruralities that involve studies on: (a) rural development; (b) political protagonism*” (Haugen et al., 2015, p. 118). The proposal for analysis that places the relationship between gender and class in the spotlight continues to be a usual conceptual path in the research of this theme; however, currently science has favored a post-structuralist perspective, transcending the classic Marxist vision: in addition to the economic sphere, the importance of gender relations is also recognized under an intersectional approach, which takes into account the individuality of these women, as well as their agricultural and community work and the analysis of the discourses of the subjects of various social spheres (Gomes et al., 2016).

With regard to studies on gender relations in the Portuguese rural space, Carmo (2007b) sought to understand how the socio-spatial divisions and social practices have changed according to gender, in the last 30 years, taking into account factors such as the modernization and urbanization of daily life in the rural community. The conclusions of this study show some reconfigurations in relation to the traditional model of division of roles, namely: the widening and intensification of social contact with the urban environment for both men and women; and the management of the domestic economy ceased to be exclusively the domain of women, with men taking on some of the burdens related to financial matters and domestic consumption (electricity, water, gas). On the other hand, the continuities of the patriarchal model of inequality still appear persistent and in large numbers. The author found that sociability relations in public contexts are still more practiced by men, as they live more regularly in urban and leisure spaces such as bars, discos, restaurants and cafés.

The appropriation of public spaces is also carried out differently according to gender differentiation, since it is the man who identifies and presents himself with a more public image, also accompanied by a greater intensity of regular social relationships (Carmo, 2007a). Added to this inequality of social capital, there are also differences in the way men and women occupy the public space during socialization: women talk more at the door of the house and in the markets while men use the café for this purpose, which reveals a continuity of the figure of the woman associated to the domestic space. The café, which used to be called “tavern,” continues to be a place described by women as awkward and where they do not feel “welcome,” because it is dominated by the opposite gender.

These customs, by allocating female power to the private and male power to the public (Lisboa et al., 2006), reproduce an asymmetry of representations and meanings attributed to these gender identities, in various dimensions of society (Amâncio, 1993, 1994), of which agricultural work is no exception. These customs, imaginaries, representations and meanings when incorporated in different spheres throughout life, define the sexual division of labor and the appropriation of certain spaces (Almeida, 2018).

Based on the findings from an exploratory qualitative study carried out in two regions of the inner Portuguese territories, we intend to explore the meanings and perceptions of women farmers on different dimensions of their professional activity. It is intended to contribute to the construction of knowledge about family farming, with special focus on its gendered dimension and the need and relevance of including women's point of view in this field of studies. Likewise, to denote the importance and need to undertake efforts to combat gender inequalities in this sector of activity, as well as to value women roles, which is so pressing in food systems worldwide. With this work, and similarly to other international theoretical approaches, we intend to unveil and understand the discourses of Portuguese women farmers, something that has not yet been presented. Our main research questions are: What are the perceptions of female family farmers toward the agricultural activity and their work? What types of discourses are dominant in the Portuguese agricultural scenario?

## Methodological approach and data collection

This work results from the project “MAIs - Women farmers in inner territories” which aims to increase the civic and associative participation of women farmers in inner Portuguese regions, through their empowerment, the promotion of the visibility of their social role and gender equality. The focus of the project is family farming, through a pilot experience in two municipalities: São Pedro do Sul and Sabugal, where the family farming population plays a relevant role. In 2019, the proportion of the family farming population was 20% in São Pedro do Sul and 30.7% in Sabugal (INE, 2021). The proportion of women in the family farming population was 49.5% in São Pedro do Sul, and, 47.4% in Sabugal, that is, almost half of the family farming population. The percentage of women farmers aged 65+ was 39% in São Pedro do Sul and 44.8% in Sabugal.

The methodological approach used in this study is qualitative, by seeking the meaning(s) and the rational adjacent to the action (Guerra, 2006), which will allow us to explore the imaginaries and symbolic universes, taking as unit of analysis the voice and discourses of the participants (Amozurrutia and Servós, 2011). The qualitative approach allows the researcher to see inside the reality at study (Lalanda, 1998), as a construction of the participants and not of the *status quo* around them (Bryman, 2016). There are several qualitative techniques, all with different functions (exploratory, analytical and expressive). Within the scope of the broader project, of which this study is part, the focus groups were carried out in an exploratory phase of research and aimed to better understand the women farmers of the municipalities in study, as well as generate and evaluate ideas collected *a priori* through theoretical analysis on the subject. They also allowed the team to facilitate access to individuals or groups normally reluctant to communicate. Its potential is to create threads of discourse, life stories and experiences that are shared during the sessions.

Focus groups (or focused group interviews) are a qualitative research technique for gathering information in which a number of people are brought together as a group to discuss a particular topic of interest, with the facilitation of a moderator who poses questions and encourages a diversity of opinions (Dawson et al., 1993). This type of group interviews is a qualitative research technique which allows for a deeper understanding and analysis of the perspective (individual and collective) of a group of selected participants about specific themes/issues (suggested by the researcher/group moderator) (Trad, 2009; Hernández-Sampieri et al., 2014; Souza, 2020).

Once the type of focus group to be conducted had been defined, the script was developed. The script was constructed with few items (less than five), in order to allow flexibility in the conduct of the focus group (recording of unforeseen but relevant themes) (Lalanda, 1998). The items considered were: (1). how agriculture entered the lives of these women; (2). understand agriculture and rural life in these regions; (3). identify perceptions about the gender division of labor; (4). understand the social/political involvement; (5). identify the main problems and needs.

Participants, identified among family farmers in both regions, were selected based on their professional activity (agriculture or livestock), gender (women) and familiarity with the issues under analysis with the aim of fostering a comfortable environment where all participants could share experiences on the topics addressed. Fourteen women farmers participated in the two focus groups (six in Sabugal and eight in S. Pedro do Sul). These participants were chosen by the officers of the Municipal Councils, and their selection, among women family farmers, was based on the availability to participate in the group interviews. Thus, this is a non-random sample by convenience. Given the characteristics of the selection method of the participants, the sample of this study is not representative of the women family farmers of the two regions, nor of the Portuguese women family farmers, therefore the results presented here are limited to this study and sample. The participants have an average age of 50.5 years old, the youngest being 28 years old and the oldest 66 years old. Half (*n* = 7) have completed secondary education and the other half has a lower level of education (*n* = 6); most are married or in a situation of cohabitation (*n* = 12), as shown in Table 1.

**Table 1:** Sociodemographic characteristics of the participants (number of participants).

**Region**		**Level of education**		**Age (years old)**		
				** < 30**	**31–60**	**>61**
Sabugal (P1 to P6)	6	Secondary education	4	1	2	1
		Basic education	2		1	2
São Pedro do Sul (P7 to P14)	8	Primary school	3			3
		Basic education	1		1	
		Secondary education	3	1	2	
		Superior education	1		1	

The two focus groups took place in May 2021, in spaces provided by the municipalities, where the conditions for the collective interviews where assured. The interviews were conducted by a member of the research project, with two people present in the room to collect information (written and recorded) and to provide logistical support if necessary. The focus groups had ~2 h, and the interviews were recorded in video to facilitate the material transcription and the identification of the speakers in moments of overlapping speeches (Bryman, 2016).

The records of the participants' speeches in the focus groups were subsequently transcribed. This analysis was focused on the excerpts of discourse considered most relevant for the objectives defined in the project. For the treatment of the qualitative data collected, a categorical content analysis approach was used. Such categorization allowed reducing the complexity of the material, identifying the main issues addressed and allowing its treatment (Guerra, 2006; Dantas, 2016), The categories were defined based on the themes contained in the guide, as well as on the strengths of the theoretical material gathered during the literature review, and on the themes that emerged during the analysis of the material.

The findings of this analysis will be presented in the next section through excerpts of the interviewees' speeches, systematized and divided according to the dimensions of analysis and corresponding categories (Guerra, 2006; Bardin, 2011). These are: perceptions of agriculture (changes in agriculture; perceptions of farming as a profession); gender division of labor in farming; perceptions about their role and difficulties as women farmers (difficulties experienced as women farmers).

## Analysis and discussion of findings

### Perceptions of agriculture

#### Changes in agriculture

The discourses of the focus group participants highlight four major changes in agriculture: corporatisation/entrepreneurship, mechanization, market competitiveness prices and community dynamics/agricultural popular economy logics.

Professionalization and the increase of companies (as opposed to family farming), together with the greater entrepreneurship in the sector, was one of the transformations identified by the participants. These transformations are associated with the bureaucratisation of agricultural activity and the aggressiveness of the market, when compared to the agriculture reality of previous generations:

“*Today's agriculture does not compare with the old days. (…) it was subsistence farming, today it's business. It's a business and there's a lot of bureaucracy along the way. (…) It requires too much documentation and paperwork (…)* **(P1, 60 years old)**

“*We are not farmers anymore; we are a company. We have accounting, we have to do invoicing, we work like a normal company.”* **(P4, 44 years old)**

With regard to mechanization, the discourses highlight the ease and time savings in the performance of certain tasks, which previously would have taken days. Improvements in the quality of life were also mentioned, as the use of machines reduced the amount of tasks with high levels of physical demand, as well as tasks that were performed with rudimentary work tools or the use of animals:

“*It completely changed (…). (…) came with tractors, machines, which in the old days was with the plow, the cows plowing the land. (…) There are machines for everything today, pretty much.”* **(P10, 66 years old)**

“*In the old days, it took almost 3 days to plow, with the help of the cows (…). It was done in stages”* **(P10, 66 years old)**

“*We loaded the pitchfork again for the cow cart. With the cow cart, we put soil in mounds (…). Now, on my farm, everything is done with a tractor. The tractor has some hooks, they take the manure and spread it and plow (…)”* **(P8, 69 years old)**

“*(…) [the use of machines] has brought us some life quality!”* **(P12, 34 years old)**

Price competitiveness, as a transformation in the sector, was mentioned. One participant highlighted the difficulty of selling on some products, especially due the lower prices on other selling platforms/markets. In other words, the monetary devaluation of certain products makes it difficult for farmers:

“*In my parents' time, if my father produced potatoes, he always sold them. If he produced rye (…) the price was always assured (…) Today, if we wanted to produce potatoes, there is no one who looks for a potato, a kilo of corn.”* **(P6, 53 years old)**

There are also several references to the changes felt in terms of community dynamics and local economy. These discourses mention the progressive abandonment of agriculture and rural territories, as well as the disappearance of community activities and forms of conviviality that are lost or replaced by other social dynamics:

“*(…) in the old days, it was family farming, it was uncles, neighbors who helped each other. Today we can't even hire a friend or relative, because if there's a problem on the farm, all we earn on our farm, is not enough to pay* [the costs of an accident, disaster, etc]*.”* **(P1, 60 years old)**

“*It was, it was… (…) a joy, it was a joy that we had there. We never did that again!* [about a community activity that was common when she was a child]” **(P4, 44 years old)**

“*It was a happier time than now. We would cut the grass, make the small bundles and put that outside to make those dolls. And then we sang, danced, it was a joy. It was happier than now.”* **(P9, 62 years old)**

The discourse of the participants allows to contextualize their view on the agricultural activity within a specific time space. This space is marked by major social transformations that the rural spaces, and more specifically the territories of the Portuguese interior, have been experiencing (Portela, 1999; Almeida, 2020). As we can see, these changes are not only limited to the professional context, but also extend beyond the social context, family dynamics and the community nature of family farming. In effect, these changes have confronted family farming with various threats and weaknesses to its continuity. Results from other works also refers to the discontinuity of the activity by household members as a result of occupations abroad, the disappearance of local markets and traditional marketing circuits, the devaluation of production in the agri-food chain, the aging of the farming population, and loss of property (Veiga, 2014).

#### Perceptions of farming as a profession

Let us now focus on the professional context of the participants and their vision in relation to their professional activity. The first idea that emerges from the participant's discourse is that agriculture is an activity opposed to following, or carrying out, studies:

“*Many say “why did you want that life?” because many studied and only I stayed in agriculture!”* **(P5, 41 years old)**

“*I was studying and when I got home from school I had a little paper saying that I should go with the cows here or there… and I took the books, I was obliged, because I didn't stay at home to study, there was no time to stay at home to study… “you take the books, put the cows away and go, you study if you want.”* **(P6, 53 years old)**

In fact, this discourse indicates that the agricultural context of the family and the lack of access to certain types of services in rural territories constitutes a constraint to follow other life paths, namely those in which an investment in formal education would be necessary. In fact, Rodrigo (1986), already have referred to traditional family farming dynamics, based on pre-determined gender roles, that restricted the woman to agricultural activity, while the man was offered more options outside this activity. On the other hand, this discourse refers to a preconception of agriculture as an activity related to low qualifications. This notion will be again mentioned in relation to life trajectories throughout the participants' relation with agriculture.

### Trajectories into farming

In relation to this category, different types of trajectories of entry into agriculture were found. Let us begin with discourses that indicate entry into the activity *via* the family:

“*I have also always worked in agriculture, since I was young, I did a bit of everything… (…) Yes, [PARENTS] were farmers and I (…) also stayed here, also a farmer (…)”* **(P9, 62 years old)**

“*I was also born there, I was born in* [Locality where she still lives] *and my parents (…) had (…) animals. And it's like that, it was through them* [the parents] *that I learned.”* **(P11, 30 years old)**

“*I always worked in agriculture, since (…) I can say almost since I was born, no, but almost. It was my parents' life, and so I continue, it has always been my life, to work in agriculture.”* **(P8, 69 years old)**

Another way of entering and remaining in agricultural activity was through marriage:

“*(…) I was studying and I would continue, it was a higher education course. Only as I got married and got that big holding…. and they said ‘you can continue and stay with this’.”* **(P1, 60 years old)**

Nevertheless, we also found participants that suggested entering into agriculture as a personal choice, either through free will or emotional reasons:

“*(…) I was not a farmer (…) it comes a little bit in the idea of me coming and learning something. I am from here (…), my husband works here (…) so, at the time (…) we decided to go ahead and do (…)we have some small plots of land that were left to themselves and we decided then to do a project, we went ahead, the project was approved (…)”* **(P12, 34 years old)**

“*I took this life option also because of my three children, because when I came here to the village, I didn't have anyone here, my parents don't live here, my in-laws, nobody. (…) this choice of life was for my children (…)”* **(P7, 40 years old)**.

The discourse analyzed also indicate pathways marked by a large volume of work and time dedicated to agricultural activity. This results in work routines that take up entire days, with uncertainty regarding overtime, causing a certain relegation of time for rest and personal life.

“*I'm up at seven in the morning every day! Sometimes it's midnight or one o'clock in the morning and I have a cow to calve and I'm there in the field accompanying her…”* **(P3, 28 years old)**

“*(…) The difficulty is that we can never schedule holidays, when we are about to leave there can be a big surprise on the farm and it all hangs around. It's positive and negative, it's always swinging. (…) schedules are difficult, with the weather and problems that arise on the farm, (…) the days are never the same, every day is different.”* **(P1, 60 years old)**

“*It's true, we don't have holidays, we don't have Saturdays, we don't have Sundays, we have to work at night, we have to work during the day… if they [cattle] remember to give birth, we have to go, they are the ones in charge (…)”* **(P4, 44 years old)**

“*(…) it's a life of hard work.”* **(P10, 66 years old)**

Moving on to the difficulties experienced within the agricultural activity, the data collected reveals difficulties at the level of marketing the production.

“*Many times we do not have market for the product… it's [a problem]. In calf rearing, there are times, when there is no market for the calves.”* **(P5, 41 years old)**

“*We never have a right price for the animals, we never have a right market for the animals (…).”* **(P4, 44 years old)**.

The uncertainties and difficulties in terms of the flow of production are further explored in other discourses. The participants' highlight the role of factors external to the activity in conditioning economic resilience:

“*(…) it's a profession of high economic risk. You're always subject to a lot of things: the weather, when the cows calve well or not. It's always a life in uncertainty (…) the income at the end of the year depends on all these factors*. ***It's always a life of great uncertainty and it takes a lot of courage to be a farmer, plus a woman****.”* **(P6, 53 years old)**

“*(…) we never know if we will harvest enough straw. It's always uncertain! Any work is always uncertain… a machine breaks down, a pipe bursts.”* **(P4, 44 years old)**

“*We only have money when we sell some calves and we only have money when we get subsidies (…)”* **(P4, 44 years old)**.

Although the difficulties and uncertainty of agricultural labor are highlighted in these interviews, these appear, in some cases, associated with a discourse that emphasizes as a positive counterpoint the independence that this activity makes possible:

“*It gives me pleasure to be a farmer, because I do what I want and what I feel like [LAUGHS]. I don't need to get up running (…) to run around. (…) we do the work, we put the hay, we put the bale (…), where we think it's best! And there is no boss “this is what you do”! It's we who decide our lives***! (P4, 44 years old)**

Although the activity, as we saw above, is marked by external difficulties, there seems to exist an intrinsic dimension that somehow compensates these extrinsic factors (independence, pleasure, freedom). In fact, the pleasure and enthusiasm, are materialized in pride, pride that the participants feel in their farm and produce, especially from the recognition and opinions of others about it:

“*I have friends from all over and when they come here they say that this meat is spectacular and for some reason it is…”* **(P3, 28 years old)**

“*I kill two calves a year and when I invite guys over to eat they say the meat is wonderful.”* **(P5, 41 years old)**

Still in this domain, some participants characterize these perceptions and feelings as key tools for success within the activity:

“*Those who go into this activity with pleasure and with love (…) that is a very great strength (…) in all the problems that arise. If it's not with their own will, they don't make it.”* **(P1, 60 years old)**

“*Those who take the farms forward, it's with pleasure and love (…). Now there are those who go there just because, but after a year they give up.”* **(P4, 44 years old)**

“*Any profession, if you don't like it, you can't do it. Now, this one is a very demanding profession.”* (**P5, 41 years old)**

This dedication is perceived as the source of energy for enduring long working routines, as well as for the necessary resilience in facing problems and difficulties and overcoming them, that are the “day to day” in agriculture. The discourse of the participants confirm that routines in agriculture are always changing, never being exactly as predicted or in the previous day (Darnhofer, 2021).

### Gender division of labor in farming

As the gender division of agricultural labor is a fundamental dimension to the study of gender issues and the understanding of existing inequalities in agricultural activity, we dedicate this section to the analysis of the discourse of the women farmers that participated on the focus groups on this topic.

Eight interviewees stress the idea that there is no inequality or difference between men and women:

“*I think… whether woman or man, each one just does what he wants. (…) [DEALING WITH CATTLE] we do everything the same, women still deal better with animals. [ON THE QUESTION OF STRENGTH] It's the same, you just have to pull for her. (…) I can stand next to the man (…) I have no fear at all.”* **(P4, 44 years old)**

“*I do everything like a man. (…) in the matter of my husband, because if he does it I do it too.”* **(P5, 41 years old)**

“*There are no differences. The differences may be in the issue of strength, but that is not relevant.”* **(P2, 62 years old)**

“*The only thing my husband doesn't do, is molding. But (…) he does everything, he peels, he waters (…)”* **(P10, 66 years old)**

One trend is clear in these discourses: in general, the participants do not identify or do not perceive situations of inequality within their work context with their partners. They rather emphasize an equity between men and women, assuming little significant differences. However, despite of it, the analysis of the discourse presents several gender differences related with gender division of agricultural labor. Let's see:

“*We have an auction park (…) if we look at the presentation of cattle, most are men. (…) it is rare for a woman to go to the cattle show, even if she is the farmer… there are one or two. I could probably list half a dozen… that's all.”* **(P6, 53 years old)**

“*Now of course, the men… they are both [THE COUPLE] farmers, the man goes.”* **(P4, 44 years old)**

“*We talk the two of us. [ABOUT THE DECISION]. It's him [THE HUSBAND]. He's more into it, than me, I'm just taking care… [OF THE ANIMALS]”* **(P5, 41 years old***)*

“*… it discards more for the man to do the business. (…) We both work, he takes care of the selling and buying and I take more care of the notes and those things… the papers. In cases, where the man has another profession, of course the woman has to take care of everything”* **(P2, 62 years old)**

These discourses highlight differences between men and women with regard to the decision-making spheres, namely in public spaces (market). The adage found in other spheres of society (Lisboa et al., 2006; Lisboa and Teixeira, 2016), is also present in the social context of these women farmers: men still dominate the formal decision-making spaces. Nevertheless, and based on the experience of some participants, decisions at the informal level are taken by them.

“*I'm the one who does the business, I'm the one who decides, although we talk to each other. We talk between the two of us, but I'm the one who says… this comes from the beginning!* **(P4, 44 years old)**

“*(…) I have a person with me [PARTNER], but he is more in another area. I'm more with cattle and sheep (…) then I'm the one who decides, I'm the one who treats, I'm the one who does the business, although I ask for his opinion (…) but I'm the one who gives the last word. (…) I don't even feel comfortable that someone else does it (…) Now the last word is mine, always.”* **(P3, 28 years old)**

“*I happen to be the one who manages the holding… what little it is [LAUGHS], I don't give it up.”* **(P6, 53 years old)**

“*He [HUSBAND] collaborates a lot, but I'm the one who takes the decisions.”* **(P1, 60 years old)**

It makes sense to find this type of independence in decision-making processes, since some of the speeches already highlighted the autonomy that the agricultural profession conferred to certain participants. However, there is a clear tendency to consult their partners before taking a decision, even though they imply that it is their word that counts the most. We thus find an inversion between formal and informal decision-making contexts, although on the informal level, the women as a protagonist is not a trend in the sample in question.

In relation to the division of tasks, between men and women, in the agricultural activity carried out by these women, some participants refer that this is carried out in an egalitarian way. Although in some cases, there are tasks that belong to each member of the couple:

“*(…) me and my [NAME OF THE PARTNER] have other professions and other things, so sometimes time management is not easy (…) one day I go, another day he goes [to the farm] (…)”* **(P12, 34 years old)**

“*Yes, he works, yes he [THE HUSBAND] also does it (…) he does it with the tractor, plows, mills, when it's the potatoes they have to go there to sow the potatoes, to help (…) he also has to go there.”* **(P9, 62 years old)**

The participants discourse also indicates situations where the woman is the only component of the couple that carries out agricultural tasks:

“*There's a cow to calve and my husband is like “never mind, it'll be fine”, but I say “we have to go watch!” and he doesn't…. And I grab the car and go there to see”* **(P1, 60 years old)**

“*(…) I do everything by myself, everything from scarifying to gardening. Everything, because I can't wait for my husband (…)”* **(P7, 40 years old)**

The discourses suggest situations of clear division of labor between the members of these couples, in which the woman takes the initiative and seems not to receive help from her partner. Also, other tasks are clearly a responsibility of the men, namely the tasks related with the use of agricultural equipment and specific tasks, usually considered to be skill demanding, such as pruning or applying pesticides/fertilizers:

“*But I see that it is like this, the man is more tractor and (…) coring and pruning (…) the woman is the one who does all the [MANUAL] work.”* **(P7, 40 years old)**

“*(…) what [P7] was saying, that men work more with machines and women don't (…) some of them are still afraid to take the tractor or to pick for the brushcutter (…) this major distinction came from that. Of the man taking the artifacts and the machines and the women working more with their hands*. **(P12, 34 years old)**

In fact, the perception that the relation with machinery and equipment belong to the men's forum, is reinforced by the participants:

“*(…) in my vineyard I do everything (…) except take the tractor.”* **(P13, 59 years old)**

“*[INTERVIEWER] (…) you don't work with the machines? [P9] No (…) I don't. Only with the hoe”* **(P9, 62 years old)**

Taking into account the participants' age, it seems that younger women are the ones who are not afraid to perform tasks using machines, compared to older women.

Still at the level of the division of tasks between men and women, some discourses refer to social imaginaries that feed differences between men and women in the performance of their activities:

“*(…) women still deal better with animals.”* **(P4, 44 years old)**

“*Men, in general, do not have the skills that women have to do several jobs at the same time… we do the house life, we still take care of this and that, but they don't! They do that and… one thing at a time (…)”* **(P14, 59 years old)**

This last discourse is of particular interest, since it denotes inequalities in the volume of work between men and women, highlighting the double working hours of women (at the farm and domestic levels).

Finally, a positive change in gender inequality within the agricultural activity is acknowledged. However, this recognition is made by women in younger age groups (between 34 and 44 years):

“*Maybe in organic farming, even in older people, there is already a greater sharing of tasks.”* **(P12, 34 years old)**

“*[ON THE FACT THAT WOMEN IMPOSE THEMSELVES ON MEN] It's like that, it's like that within my age group. Now in the older ones, maybe not…, but the younger ones, we are already more resourceful (…)”* **(P4, 44 years old)**

“*(…) As a rule, now there is not so much difference… women, we impose ourselves and that's it [LAUGHS]”* **(P4, 44 years old)**

### Perceptions about their role and difficulties as women farmers

Finally, and because this appears to be an extremely important dimension of analysis in the study in question, we will analyse the perceptions about their role as women farmers, either by themselves or by others.

The interviewees reported discrediting by men, which arises in comments, based on asymmetries in representation between the masculine and feminine. Other works related with gender divisions and agriculture, these social representations construct gender, and, when they are asymmetrical, they dictate not only a division of labor, but also gender inequality (Amâncio, 1994). Once again, it is possible to verify the social perception of agriculture as a male activity, based on socially shared ideas resulting from social representations of gender. These social representations are reflected in the strangeness of being a woman performing tasks, or occupying spaces intended for men.

“*(…) just because we are women, I think it's a discrediting of our work. Why? Ah, she's a woman, she doesn't have as much strength as a man (…) she won't be able to do it (…). When I started, they said I couldn't stand it or manage, just because I was alone and couldn't carry out the activity. [INTERVIEWER]: And did you hear that (…) from family, friends? [P3] In general (…) from the male person, (…) first of all bearing in mind the age at which I started (…) why I took up farming and the fact that I was so young, that I was a woman and they said that I couldn't carry out the activity as well as a man.”* **(P3, 28 years old)**.

“*When I had the sheep and when we started to sell the cattle, my husband wasn't there because the sheep were mine, my husband had his profession. And the guy [BUYER] arrive there like ‘then your husband isn't here'? So, now how can I buy the lambs from him?' (…) I was like “then if you have to buy them, you'll buy them from me”; [BUYER BY THE VOICE OF THE WOMEN] “and you sell me the lambs? (…) and then your husband?” [P4 REFERRING TO HER OWN VOICE] “My husband doesn't have anything, so, I mean, this is mine, is he the one in charge now? (…)” (…)* ***men respect a man more than a woman… all my life it was like that*.**” **(P4, 41 years old)**

## Conclusions

The exploratory data we present suggest that these group of Portuguese women farmers, from the inner territories, work in a social context that has undergone major changes over the last decades, whose impact is referred to in the discourses at the level of agricultural practices and activities, marketing of products and in community dynamics. In several cases, the trajectories of life in agriculture started very early, and the permanence in the activity was the result of external influence: family or marriage. On the other hand, the agricultural work of these two groups of women, as well as their own activity, is characterized by long, solitary and uncertain working days, that they value due to intrinsic dimensions, mainly the results of production, independence regarding the organization of the work or the possibility (idea) of making their own decisions.

Also in the gender dimension, the public/private dichotomy is clear, especially in decision-making places: formal and public places are dominated by men; informal and within the couple (private) are equal places, or dominated by the woman farmer, often with consultation with the male partner. Gender is also a reason for discrediting their abilities as women farmers, in the perception of some of the participants, making their activity even more difficult.

The speeches of the interviewed women are compatible with the three types of discourses identified by Brandth (2002). The premise of the first discourse that portrays the male farmer as the “public figure” of the family still holds true, an example of which is pointed out in reference to cattle marketing spaces. We also perceive that the agricultural tasks dominated by the man are the most mechanized ones, since many of the female farmers do not know how to operate the machinery and in their discourse assume these tasks as being masculine (presence of the discourse of “masculinization”). Manual work is still performed by women farmers, which is related with the family-based and small scale portrait. The discourse “detraditionalization and diversity” is also present in younger women farmers (34 and 40 years old), that choose agriculture as a profession, and invested in agricultural projects, and want an healthy and sustainable life for their families.

With regard to the analysis of gender relations, lack of time and the need to attend to domestic responsibilities are the main obstacles that prevent them from participating in social and economic activities. This is aligned with other authors, such as Gomes et al. (2016) that mentioned that women's patterns of displacement and participation in activities stem from a demarcation of “gender roles,” which still remain traditional and restricted to the private space of the home.

The discourses presented are not enough to allow wider deepening of some life dimensions of these women farmers, in particular the life trajectories and other dynamics related to gender roles and representations, with special focus on decision-making places. Also, the limits of the information collection technique become clear throughout the exposition of the data, namely the strong participation of some interviewees in relation to others, and reduced approaches to certain topics (Bryman, 2016). In terms of limitations in the present sample, the representation of women from older age groups, influences the perceptions about the changes in agriculture, that are usually considered to have more knowledge on this topic. Probably, an effort of the moderators to encourage the participation of younger women in these interview topics, would allow to collect information to compare meanings and perceptions. These limitations can be compensated in future research in these areas, articulating the participation of younger women with older women, and thus promoting dialogues and data for intergenerational comparison.

However, given the exploratory richness of the material, we believe that these offer new evidence and clues for future research and work in these meanderings, particularly at the Portuguese level, with special focus on portraits of the figure of the women in the family farming context in the first person. By making this undertaking, we will contribute to the construction of new knowledge on forms of gender inequality which have not been the target of intervention - making the invisible “visible” is the first step toward what follows: *changing this invisible*.

Several international organizations recognize the fragility to which women farmers are subjected, while also recognizing the benefits, at local and global levels, that arises from the promotion of gender equality in family farming holds (FAO, 2011, 2014; EIGE, 2016), since we are dealing with the most common type of agriculture, that contributes with undeniable importance for the world food systems and for the fight against malnutrition and hunger. Also, it is widely recognized that this sector support sustainable agricultural systems, mainly based on traditional and ecological practices (Costa et al., 2018; Aguiar et al., 2020), thus being pathways toward the Sustainable Development Goals and against the current climate crisis.

In fact, the profound transformations of global realities such as agriculture can no longer be analyzed only from one dimension, but rather as a result of the intersection of different perspectives (technical, social, economic, environmental, among other), challenging disciplinary boundaries and taking into account all the faces of a phenomenon. Only by doing so, it is possible to promote social wellbeing and contribute to the eradication of inequality.

## Data availability statement

The raw data supporting the conclusions of this article will be made available by the authors, without undue reservation.

## Ethics statement

The studies involving human participants were reviewed and approved by COMISSÃO DE ÉTICA DO POLITÉCNICO DE VISEU. The patients/participants provided their written informed consent to participate in this study.

## Author contributions

Conceptualization: CC and MJ. Methodology: CC, RR, and DG. Validation: RR. Field work: DG, CC, and CB. Formal analysis and investigation: MJ, DG, and CB. Resources, data curation, and project administration: CC. Writing—original draft preparation: DG and MJ. Writing—review and editing: DG, MJ, and CC. Supervision and funding acquisition: CC and RR. All authors contributed to the article and approved the submitted version.

## Footnotes

^1^It is important to note that the feminization of agriculture is not a linear process. Different geo-spatial contexts explain different paths for the feminisation of agriculture, coupled with different broad social transformations. Some authors, as Cernea (1978) and Lastarria-Cornhiel (2008) refer different factors leading to the feminisation of agricultural assets, in contexts outside the Portuguese case.
